# Efficient PPA-SiO_2_-catalyzed Synthesis of β-enaminones Under Solvent-Free Conditions

**DOI:** 10.3390/molecules181215182

**Published:** 2013-12-10

**Authors:** Muhammad Nisar, Ihsan Ali, Muhammad Raza Shah, Mughal Qayum, Muhammad Zia-Ul-Haq, Umer Rashid, Md. Saiful Islam

**Affiliations:** 1 Institute of Chemical Sciences, University of Peshawar, Peshawar-25120, Pakistan; E-Mail: ihsanali7@yahoo.com; 2 H.E.J. Research Institute of Chemistry, International Center for Chemical and Biological Sciences, University of Karachi, Karachi 75270, Pakistan; E-Mail: raza_shahm@yahoo.com; 3 Department of Pharmacy, Kohat University of Science and Technology, Kohat-26000, Pakistan; 4 The Patent Office, Karachi 74400, Pakistan; E-Mail: ahirzia@gmail.com; 5 Institute of Advanced Technology, Universiti Putra Malaysia, 43400 UPM Serdang, Selangor, Malaysia; 6 Department of Chemistry, Faculty of Science, Universiti Putra Malaysia, 43400 UPM Serdang, Selangor, Malaysia; E-Mail: mdsaifulislam@upm.edu.my

**Keywords:** solvent free, 5,5-dimethylcyclohexan-1,3-dione, PPA-SiO_2_, β-enaminones

## Abstract

An efficient method has been developed for the synthesis of β-enaminones under solvent-free reaction conditions using PPA-SiO_2_ as catalyst. The reaction yields were good to excellent (up to 90%). This methodology affords high selectivity and good tolerance of a variety of different functional groups present on both aromatic and aliphatic amines. In addition, the methodology is environmentally benign and cost-effective due to absence of solvent and easy work-up.

## 1. Introduction

Enaminones are chemical compounds including an amino group coupled through a C=C bridge to a carbonyl group. They are versatile synthetic intermediates [1,2,3] that combine the ambident electrophilicity of an enone with the ambident nucleophilicity of an enamine. They be easily synthesized and purified because the carbonyl group conjugates with the enamine therefore, imparts an additional stability to the enaminone motif [1].

Enaminones are important building blocks in organic synthesis which can be further transformed into valuable bioactive nitrogen heterocylces [4,5,6], natural therapeutic agents and alkaloids [5,6]. They are also used as precursors for the synthesis of various types of compounds like N-substituted carbazolones [7], peptides [8], quinolines [9,10], azocompounds [11,12], α,β-aminoacids [13,14] that serve important roles in asymmetric catalysis via chelating agents [15]. Enaminones are known to affect several physiological functions themselves or are precursors for such molecules, in particular as anticonvulsant [16], anti-epileptic [17], anti-inflammatory [18] and antitumor agents [19,20,21].

Owing to the significant role of enaminones in organic synthesis considerable efforts have been made for their synthesis. The most straightforward route is the direct condensation of the 1,3-dicarbonyl with an amine under reflux with azeotropic removal of the water formed [22]. Some improved methods subsequently reported for this transformation are SiO_2_/microwaves [23], NaAuClO_4_ [24], iodine [25], HClO_4_·SiO_2_ [26], SiO_2_-sulphuric acid [27], SiO_2_-NaHSO_4_ [28], tris(hydrogensulfato) boron or trichloroacetic acid [29], ionic liquids [30], and silica-supported Fe(HSO_4_)_3_ [31]. Other synthetic approaches that lead towards β-enaminones are the reductive cleavage of isoxazoles [29], the use of expensive catalysts like [BMIM]BF_4_, cyclization of amino acids [32], and copper-mediated aminolysis of dithioacetals [33]. However, some of these methods suffer from certain drawbacks such as formation of side products, low selectivity and use of toxic reagents and solvents.

Over the past few decades, solid-supported catalysts have been viewed as unique acid catalysts in the sense that the effective surface area of the active phase can be increased many-fold, since the activity and selectivity of the precursor center dispersed on the surface of support is improved. Polyphosphoric acid (PPA) absorbed on silica gel is certainly one of the green catalysts that is easily prepared and have been used for various organic transformations [34].

In continuation of our interest toward the development of new protocols [35], we report herein that PPA-SiO_2_SiO_2_ can be used as a cheap and efficient catalyst for the synthesis of β-enaminones under solvent free conditions by direct transformation of 1,3-dicarbonyls with various aromatic and aliphatic amines. To the best of our knowledge the use of PPA-SiO_2_ as an efficient catalyst for the synthesis of biologically and synthetically significant β-enaminones has not been previously reported.

## 2. Results and Discussion

The reaction conditions were optimized by taking the reaction between 1,2-diaminomethane-phenylene and dimedone as model reaction. Initially, the model reaction was carried out without catalyst in ethanol at reflux for 2 h and only 30% of the desired product was obtained. (Table 1, Entry 1). The model reaction was carried out in protic solvents (like H_2_O, CH_3_OH and C_2_H_5_OH) and aprotic solvents (like CH_3_CN, CHCl_3_ and tetrahydrofuran (THF)) using PPA-SiO_2_. Considering the yields, methanol and ethanol were found to be the best solvents (Table 1). However, when the reaction was carried out under solvent free condition, both yield and reaction time were significantly improved at about 70 °C as compared to the reaction carried out in solution (Table 1, Entry 8).

**Table 1:** Comparative study of the solvent *versus* solvent free reaction conditions for enaminones. 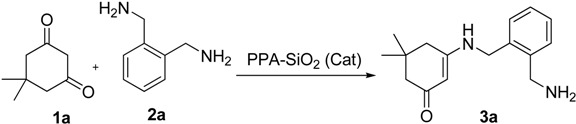

Entry ^a^	Solvent	Catalyst	Temperature (°C)	Time (min)	Yield ^b^ (%)
1.	Ethanol	−	Reflux	4h	30
2.	Water	PPA-SiO_2_	80	90	Traces
3.	Methanol	PPA-SiO_2_	Reflux	130	70
4.	Ethanol	PPA-SiO_2_	Reflux	140	71
5.	CH_3_CN	PPA-SiO_2_	Reflux	140	48
6.	Chloroform	PPA-SiO_2_	Reflux	130	56
7.	THF	PPA-SiO_2_	Reflux	140	Traces
**8.**	**Solvent free**	PPA-SiO_2_	**70**	**40**	**88**

^a^ *Reaction conditions*: see typical procedure; PPA-SiO_2_ 110 mg/mmol; ^b^ Isolated yield.

### 2.1. Effect of Loading Catalyst

In order to optimize the amount of PPA-SiO_2_ used as catalyst, the model reaction was carried out for the formation of desired product with varying amounts, *i.e.*, 50, 80, 110, 130 and 150 mg. It was observed that the rate of reaction and yield of the product increased with the increase of catalyst load. The optimum amount of catalyst turned out to be 110 mg/mmol. Above 110 mg the catalyst showed no significant effect on the yield of products (Table 2).

**Table 2:** Effect of PPA-SiO_2_ as catalyst, loading for the synthesis of β-enaminones (**3a**).

Entry ^a^	Catalyst (mg)	Time (min)	Yield ^b^ (%)
1.	50	60	50
2.	80	50	64
3.	**110**	**40**	**89**
4.	130	45	86
5.	150	45	86

^a^ *Reaction conditions*: see typical procedure; ^b^ Isolated yield.

### 2.2. Amines Substrate Scope

**Scheme 1:**
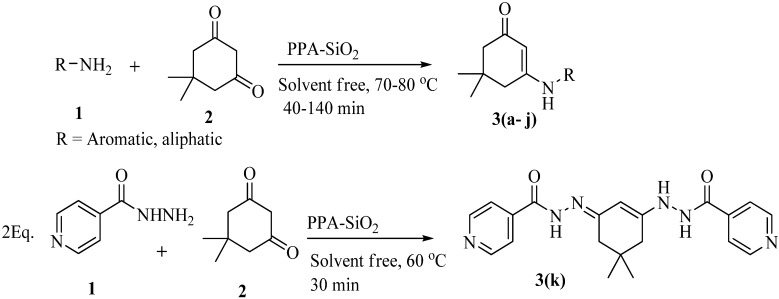
Synthetic pathway for the preparation of β-enaminones.

The substrates were evaluated for the effects of different substituents (both aromatic and aliphatic amines) on the β-enaminone analogues. The formation of desired products was investigated as shown in Scheme 1. It was observed that the reaction conditions tolerate many functional groups, including electron withdrawing (nitro), electron donating (methoxy) and halides functions on the aromatic moiety as well as in aliphatic amines. The aromatic amines were found to be more reactive than aliphatic amines (Table 3).

**Table 3:** Synthesis of β-enaminones (**3a**–**k**) in the presence of PPA-SiO_2_ as catalyst.

Entry ^a^	R	Time (min)	Product	Yield ^b^ (%)	Observed mp (°C)	Lit. mp (°C)
1.	1,2-Phenylene -dimethanamine	40	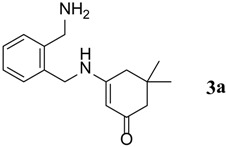	89	187–188	186 [30]
2.	C_6_H_5_-	40	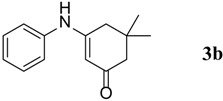	68	152–154	−
3.	4-OCH_3_C_6_H_5_-	45	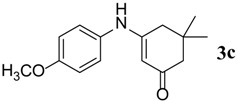	74	201–202	199 [34]
4.	4-CH_3_C_6_H_5_-	40	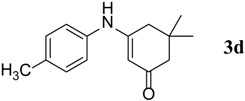	74	201–202	199 [34]
5.	4-BrC_6_H_5-_	40	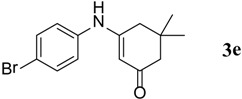	88	215–217	219 [32]
6.	4-NO_2_C_6_H_5_	50	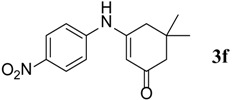	67	194–195	191 [32]
7.	(1S,2S)-(+)1-Amino-1-phenyl-1,3-proanediol	60	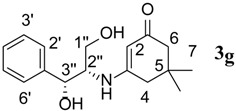	71	103–105	−
8.	D-Threo-2-amino-1-(4-nitrophenyl)-1,3-propanediol	60	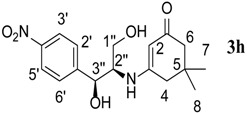	70	176–178	−
9.	4-(methyl)cyclohexane carboxylic acid	140	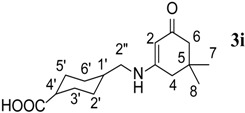	48	270–272	−
10.	2-(1-(methyl)cyclohexyl)acetic acid	145	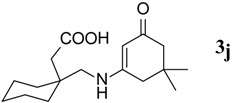	50	155–158	−
11.	Isonicotinic acid Hydrazide	30	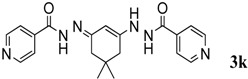	90	181–183	−

^a^ *Reaction conditions*: all the reaction were carried under solvent free conditions by heating the reaction flask in an oil bath at 70–80 °C, and withg 110 mg/mmol of PPA-SiO_2_ as catalyst. The reaction progress was monitored by TLC (ethyl acetate: *n*-hexane 5:1); ^b^ Isolated yield.

After the successful completion of the synthesis of β-enaminone analogues we proceeded to study the reaction of various amines both aliphatic and aromatic under solvent free conditions. The study was extended to explore the reaction of isonicotinohydrazide. Variation in the product was observed when isonicotinohydrazide was made to react with dimedone (Entry 11, Table 3), and N'-(3-(2-isonicotinoylhydrazinyl)-5,5-dimethylcyclohex-2-enylidene)isonicotino-hydrazide (**3k**) was obtained in 90% yield within 30 min. The variation in product is due to the increased reactivity of this hydrazide.

### 2.3. Proposed Mechanism

**Scheme 2:**
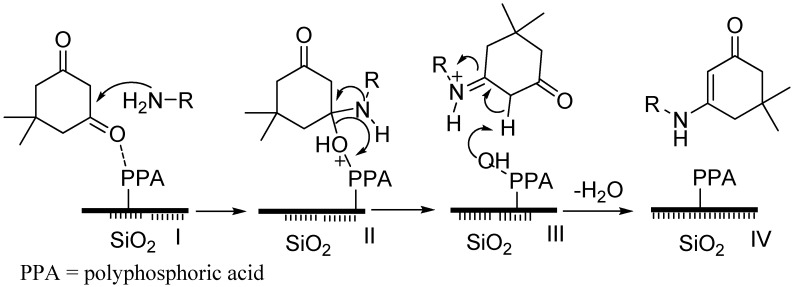
Proposed mechanism for the formation of β-enaminones.

The catalyst PPA-SiO_2_ activates the ketonic carbon for nucleophilic attack on the amino group. The active species in PPA-SiO_2_-catalyzed synthesis of β-enaminones are the amino-alcohol and imine intermediates. Finally, the target product β-enaminone would be released on dehydration of the imine intermediate. The proposed mechanism for the formation of β-enaminones is given in Scheme 2.

## 3. Experimental

### 3.1. General

The commercially available solvents used were purified by using standard purification methods. The other reagents and amines were purchased and used as received without further purification. The chiral amines (1*S*,2*S*)-(+)-1-amino-1-phenyl-1,3-proanediol and D-threo-2-amino-1-(4-nitrophenyl)-1,3-propanediol used were Sigma-Aldrich (St. Louis, MO, USA). These amines are single isomers having fixed stereochemistry and therefore give single products without any ambiguity. The NMR spectra of the products were recorded using a Bruker Avance spectrometer (Bruker, Karlsruhe, Germany) operating at 300 or 400 MHz for the ^1^H-NMR spectra and 75 or 100 MHz for ^13^C-NMR spectra. CDCl_3_ was used as an NMR solvent and TMS was taken as internal standard. The compounds were also analyzed by mass spectra (EI-MS) spectroscopic analysis, performed on a Finnigan-MAT-311-A apparatus (Finnigan MAT, Waltham, CA, USA) and the values were reported in *m/z* (rel. abund.%). CHN analysis was carried out by using Carlo Erba Strumentasion-Mod-1106, Milan, Italy. Melting points of the synthesized compounds were determined with Electrothermal melting point apparatus (Essex, UK) and are uncorrected. IR spectroscopic analysis was performed on Shimadzu-IR-460 (Shimadzu Corporation, Kyoto, Japan) for neat samples. Isolated yields refer to the amount obtained either by recrystalization from a suitable binary solvent system or column chromatography on silica gel (200–300 mesh), using a mixture of ethyl acetate and *n*-hexane as eluent.

### 3.2. Preparation of PPA-SiO_2_ Catalyst

PPA-SiO_2_ was prepared by the reported method [23] with slight modifications. A round-bottom flask already dried at 120 °C was charged with a magnetic bar, polyphosphoric acid (2.5 g) and CHCl_3_ (120 mL) and the mixture was stirred for 1 h at 50 °C. SiO_2_ (100–200 mesh, 5.1 g) was added and the stirring was continued for another 1 h. The excess of chloroform was evaporated under vacuum using rotary evaporator. The solids obtained were dried under vacuum oven for 4 h at 120 °C.

### 3.3. Typical Procedure for PPA-SiO_2_ Catalyzed Synthesis of β-Enaminones

To a mixture of dicarbonyl compound (dimedone, 1 mmol, 140 mg) and 1,2-diaminomethane-phenylene (1 mmol, 0.14 mL) PPA-SiO_2_ (110 mg/mmol) was added in in one lot and the resulting yellow mixture was stirred at 70–80 °C for the specified time shown in the Table 3. After the completion of reaction or sufficient amount of product formation (indicated by TLC) the reaction mixture was cooled and the yellow product slurry was collected by filtration on dilution with ethyl acetate or dichloromethane. The insoluble catalyst was separated by filtration and concentrated under reduced pressure. The cured product obtained was purified in most cases by recrystallization in binary solvent system using EtOH-H_2_O (2:1), while some of the product (in case of solid amines) was also purified by using column chromatography. The structures and purity of all of the products were confirmed by NMR (^1^H-NMR and ^13^C-NMR) EIMS, IR and CHN analysis. The data for new representative examples are listed below:

*3-(2-(Aminomethyl)benzylamino)-5,5-dimethylcyclohex-2-enone* (**3a**). ^1^H-NMR (CDCl_3_, 300 MHz) δ = 8.31 (s, 2H, NH_2_), 8.41 (brs, 1H, NH), 7.08–7.04 (m, 4H, Ar-H), 4.96 (s, 1H, CH), 4.14 (s, 2H, CH_2_-NH_2_), 2.14 (s, 2H, CH_2_), 2.03 (s, 2H, CH_2_), 0.93 (s, 6H, 2CH_3_); ^13^C-NMR (CDCl_3_, 75 MHz) δ = 197.8 (C=O), 165.1 (-NH-C=), 137.3, 128.0, 127.7, 127.3, 126.5 (aromatic carbons), 94.9 (C=CH), 48.9 (CH_2_), 46.5 (CH_2_), 44.1 (CH_2_), 42.7 (CH_2_), 32.6 (2CMe_2_), 27.9 (2CH_3_); IR (neat): ʋ 3,288, 1,650, 1,593, 1,537, 1,234, 790 cm^−1^; EIMS *m/z* (%): 258.15 [M^+^+H], (8.51). Anal. Calcd. for C_16_H_22_N_2_O: C, 74.38; H, 8.58; N, 10.84. Found: C, 74.45; H, 8.50; N, 10.89.

*3-((1R,2S)-1,3-Dihydroxy-1-phenylpropan-2-ylamino)-5,5-dimethylcyclohex-2-enone* (**3g**). ^1^H-NMR (400 MHz, CDCl_3_) δ = 7.37–7.19 (m, 5H, Ar-H), 6.34 (brs, 1H, NH), 5.80 (s, 1H, CH, C-2), 5.18 (s, 1H, OH), 5.07 (s, 1H, OH), 4.13–3.81 (m, 4H, C-1'', C-2'', 3''), 2.47–2.21 (m, 4H, 2CH_2_, C-2,6), 0.94 (s, 3H, CH_3_), 0.67 (s, 3H, CH_3_); ^13^C NMR (100 MHz, CDCl_3_) δ = 196.8 (C=O), 163.1 (NH-C=), 148.4, 147.1, 139.5, 138.5, 132.5, 130.0 (aromatic carbons), 102.2 (C=CH), 68.1 (CH-OH), 62.1 (CH-NH), 60.7 (CH_2_-OH), 49.7 (CH_2_), 42.3 (CH_2_), 33.6 (2CMe_2_), 27.3 (CH_3_), 26.5 (CH_3_); IR (neat): ʋ 3,608, 3,540, 3,214, 3,018, 2,924, 1,648, 1,559, 1,229, 739 cm^−1^; EIMS *m/z* (%): 290.0 [M^+^+H], (74.9). Anal. Calcd. for C_16_H_22_NO_3_: C, 69.54; H, 8.02; N, 5.07. Found: C, 69.60; H, 8.05; N, 5.10.

*3-((1S,2R)-1,3-Dihydroxy-1-(4-nitrophenyl)propan-2-ylamino)-5,5-dimethylcyclohex-2-enone* (**3h**). ^1^H-NMR (400 MHz, CDCl_3_) δ = 8.11 (d, *J* = 8.8 Hz, 2H, Ar-H, C-2',6') 7.47 (d, *J* = 8.8 Hz, 2H, Ar-H, C-3'-5'), 5.12 (s, 1H, CH, C-2), 3.06 (s, 2H, OH), 3.30 (t, *J* = 2.8 Hz, 2H, C-3'', 2'') 3.12 (s, 2H, C-1'') 2.15 (d, *J* = 16.0 Hz, C-6), 2.01 (d, *J* = 17.2 Hz, C-4), 0.92 (s, 3H, CH_3_), 0.77 (s, 3H, CH_3_); ^13^C-NMR (100 MHz, CDCl_3_) δ = 197.1 (C=O), 165.6 (NH-C=), 149.4, 147.1, 126.5, 123.3 (aromatic carbons), 94.3 (C=CH), 69.15 (CH-OH), 60.1 (CH-NH), 59.5 (CH_2_-OH), 48.7 (CH_2_), 43.3 (CH_2_), 32.6 (2CMe_2_), 27.9 (2CH_3_), 27.5 (2CH_3_); IR (neat): ʋ 3,590, 3,540, 3,198, 3,010, 2,952, 1,644, 1,589, 1,249, 719 cm^−1^; EIMS *m/z* (%) 335.1 [M^+^+1], (20.8): Anal. Calcd. for C_17_H_22_N_2_O_5_: C, 61.07; H, 6.63; N, 8.38. Found: C, 61.01; H, 6.59; N, 8.43.

*4-((5,5-Dimethyl-3-oxocyclohex-1-enylamino)methyl)cyclohexanecarboxylic acid* (**3i**). ^1^H-NMR (400 MHz, CDCl_3_) δ = 6.93 (s, 1H, NH); 4.76 (s, 1H, CH), 2.82–2.78 (m, 4H, 2H, 2CH_2_), 2.16 (m, 2H, CH_2_NH), 2.10 (m, 1H, CH, C-4'), 1.93 (m, 4H, 2CH_2_, C-3', C-5'), 1.77, 1.31 (m, 2H, CH_2_, C-6'), 1.47, (m, 1H, C-1'), 1.27, 0.94 (m, 2H, CH_2_, C-2'), 0.91 (s, 3H, CH_3_), 0.87 (s, 3H, CH_3_); ^13^C-NMR (CDCl_3_, 100 MHz) δ = 193.6 (C=O), 176.7 (Carboxylic acid carbon), 162.9 (NH-C=), 93.6 (C=CH), 50.2 (CH_2_-C=O), 48.2 (CH_2_-NH), 42.5 (CH), 42.0 (CH_2_), 38.7 (CH), 35.5 (CHCOOH), 32.1 (2CMe_2_), 29.5 (CH_2_), 27.9 (CH_3_), 27.8. (CH_3_). IR (neat): ʋ 3,251, 2,937, 1,683, 1,516, 1,240, 1,149, 769 cm^−1^; EIMS *m/z* (%): 279.1 [M^+^+1], (53.5). Anal. Calcd. for C_16_H_25_NO_3_: C, 68.79; H, 9.02; N, 5.01. Found: C, 68.75; H, 9.00; N, 5.10.

*2-(1-((5,5-Dimethyl-3-oxocyclohex-1-enylamino)methyl)cyclohexyl)acetic acid* (**3j**). ^1^H-NMR (400 MHz, CDCl_3_) δ = 7.52 (brs, 1H, NH), 5.43 (s, 1H, CH), 3.19 (s, 2H, CH_2_NH), 2.45 (s, 2H, CH_2_), 2.31 (s, 2H, CH_2_), 2.20 (s, 2H, CH_2_), 1.4–1.3 (m, 10H, 5CH_2_), 1.05 (s, 3H, CH_3_), 1.03 (s, 3H, CH_3_); ^13^C-NMR (100 MHz, CDCl_3_) δ = 195.5 (C=O), 175.9 (COOH), 168.5 (NH-C=), 109.5 (C=CH), 51.6 CH_2_), 48.1 (CH_2_NH), 43.8 (CH_2_), 40.6 (C-3'') 32.7 (2CMe_2_), 28.3, 28.0 (2CH_3_), 25.4 (C-6', C-2') 22.6, 21.4, (C-4', 5',3'). IR (KBr): ʋ 2,927, 2,848, 1,708, 1,643, 1,581, 1,373, 1,247, 1,143 cm^−1^; EIMS *m/z* (%): 293.30 [M^+^+H] (13.7). Anal. Calcd. for C_17_H_27_NO_3_: C, 69.59; H, 9.28; N, 4.77. Found: C, 69.55; H, 9.20; N, 4.70.

*(E)-N'-(3-(2-Isonicotinoylhydrazinyl)-5,5-dimethylcyclohex-2-enylidene)isonicotinohydrazide* (**3k**). ^1^H-NMR (400 MHz, CDCl_3_) δ = 10.84 (brs, 2H, NH), 8.52 (d, *J* = 5.6 Hz, 4H, Ar-H), 7.76 (d, *J* = 6 Hz, 4H, Ar-H), 5.81 (s, 1H, CH), 2.40 (s, 4H, 2CH_2_), 1.04 (s, 6H, 2CH_3_). ^13^C-NMR (100 MHz, CDCl_3_) δ = 164.8 (2NH-C=O), 149.7 (NH-C=), 121.7, (aromatic carbons), 90.1 (C=CH), 48.9 (CH_2_), 40.3 (CH_2_), 32.2 (2CMe_2_), 27.4 (2CH_3_); IR (KBr) ʋ 2,938, 2,889, 1,674, 1,558, 1,481, 1,367, 1,261, 1,147, 698 cm^−1^; EIMS *m/z* (%): 378.1 [M^+^+H), (100.0). Anal. Calcd. for C_20_H_22_N_6_O_2_: C, 63.48; H, 5.86; N, 22.21. Found: C, 63.40; H, 5.80; N, 22.18.

## 4. Conclusions

In conclusion, we have successfully developed an efficient strategy for the synthesis of novel and known β-enaminone analogues by the treatment of various aromatic and aliphatic amines with a 1,3-dicarbonyl compound (dimedone) in the presence of PPA-SiO_2_ under solvent free conditions. This methodology showed a wide substrate scope for amines (both aliphatic and aromatic). The advantages of this acceptable protocol are chemoselectivity, short reaction times, cleaner reaction profiles, simplicity, low cost, high yield and finally accordance with the green chemistry protocols. It is proposed that the methodology can be utilized for large-scale ecofriendly preparation of such a synthetically important class of compounds. Further exploration of the biological applications of the presented compounds is under investigation in our laboratory.
